# High-throughput prediction of enzyme promiscuity based on substrate–product pairs

**DOI:** 10.1093/bib/bbae089

**Published:** 2024-03-14

**Authors:** Huadong Xing, Pengli Cai, Dongliang Liu, Mengying Han, Juan Liu, Yingying Le, Dachuan Zhang, Qian-Nan Hu

**Affiliations:** CAS Key Laboratory of Computational Biology, CAS Key Laboratory of Nutrition, Metabolism and Food Safety, Shanghai Institute of Nutrition and Health, University of Chinese Academy of Sciences, Chinese Academy of Sciences, Shanghai 200031, China; CAS Key Laboratory of Computational Biology, CAS Key Laboratory of Nutrition, Metabolism and Food Safety, Shanghai Institute of Nutrition and Health, University of Chinese Academy of Sciences, Chinese Academy of Sciences, Shanghai 200031, China; CAS Key Laboratory of Computational Biology, CAS Key Laboratory of Nutrition, Metabolism and Food Safety, Shanghai Institute of Nutrition and Health, University of Chinese Academy of Sciences, Chinese Academy of Sciences, Shanghai 200031, China; CAS Key Laboratory of Computational Biology, CAS Key Laboratory of Nutrition, Metabolism and Food Safety, Shanghai Institute of Nutrition and Health, University of Chinese Academy of Sciences, Chinese Academy of Sciences, Shanghai 200031, China; Institute of Artificial Intelligence, School of Computer Science, Wuhan University, Wuhan 430072, China; CAS Key Laboratory of Computational Biology, CAS Key Laboratory of Nutrition, Metabolism and Food Safety, Shanghai Institute of Nutrition and Health, University of Chinese Academy of Sciences, Chinese Academy of Sciences, Shanghai 200031, China; Institute of Environmental Engineering, ETH Zurich, Laura-Hezner-Weg 7, 8093 Zurich, Switzerland; CAS Key Laboratory of Computational Biology, CAS Key Laboratory of Nutrition, Metabolism and Food Safety, Shanghai Institute of Nutrition and Health, University of Chinese Academy of Sciences, Chinese Academy of Sciences, Shanghai 200031, China

**Keywords:** enzyme screening, enzyme promiscuity, deep learning, substrate-product pair, web server

## Abstract

The screening of enzymes for catalyzing specific substrate–product pairs is often constrained in the realms of metabolic engineering and synthetic biology. Existing tools based on substrate and reaction similarity predominantly rely on prior knowledge, demonstrating limited extrapolative capabilities and an inability to incorporate custom candidate-enzyme libraries. Addressing these limitations, we have developed the Substrate–product Pair-based Enzyme Promiscuity Prediction (SPEPP) model. This innovative approach utilizes transfer learning and transformer architecture to predict enzyme promiscuity, thereby elucidating the intricate interplay between enzymes and substrate–product pairs. SPEPP exhibited robust predictive ability, eliminating the need for prior knowledge of reactions and allowing users to define their own candidate-enzyme libraries. It can be seamlessly integrated into various applications, including metabolic engineering, *de novo* pathway design, and hazardous material degradation. To better assist metabolic engineers in designing and refining biochemical pathways, particularly those without programming skills, we also designed EnzyPick, an easy-to-use web server for enzyme screening based on SPEPP. EnzyPick is accessible at http://www.biosynther.com/enzypick/.

## INTRODUCTION

Enzymatic transformations are instrumental in many fields, including the biosynthesis of bulk and fine chemicals and the biodegradation of toxic and harmful substances [1–4]. A crucial prerequisite for these applications is the discovery of functional enzymes for given reactions, particularly for the design and experimental implementation of new biosynthetic pathways [5–7]. Currently, the selection of candidate enzymes mainly relies on sequence homology, reaction similarity, and other specific characteristics. While sequence similarity is frequently employed for selecting candidate enzymes, the correlation between sequence similarity and function is not always perfectly aligned [8–10]. This inconsistency necessitates the use of delicate bioinformatics pipelines that consider other variables when selecting enzyme sequences. For instance, Mak *et al*. [11] utilized a combination of bioinformatics and molecular modeling to explore sequence databases to select a diverse panel of enzymes capable of catalyzing a targeted reaction. Similarly, Carbonell *et al*. [12] employed a candidate-enzyme scoring approach that considers sequence homology and reaction similarity. However, these workflows often involve intricate bioinformatics pipelines and are not readily accessible through web interfaces. To bridge this gap, enzyme-screening tools have been developed, such as PU-EPP [13], Selenzyme [14], E-zyme2 [15] and the ELP model [16], to enable researchers who are not bioinformatics specialists to participate in enzyme selection and to integrate these tools into other workflows [17–20]. Despite these advancements, most enzyme-screening tools are still based on reaction similarity, and their outputs are typically Enzyme Commission (EC) or Kyoto Encyclopedia of Genes and Genomes (KEGG) Orthology numbers rather than specific enzymes [21]. An EC number can represent a vast array of sequences. For instance, there were as many as ~60,000 amino acid sequences for EC1.1.1.1 in UniProt [22]. This vastness makes it challenging to prioritize a manageable number of target sequences for experimental testing. Although Selenzyme 2.0 provides amino acid sequences and multiple selection scores [23], it faces challenges when dealing with incomplete or non-comparable reactions, such as orphan reactions. In addition, the inability of current tools to accept and utilize custom enzyme libraries restricts the coverage of enzyme screening. Therefore, an innovative solution is urgently required to overcome these limitations and challenges.

In recent years, the ever-expanding reservoir of biological data and advancements in deep learning have transformed the approach to biological problem-solving [24–28]. Machine-learning-based methods have been increasingly deployed for enzyme-related tasks [25], such as prediction of EC numbers [29], family-wide enzyme-substrate specificity screening [30, 31], automatic enzyme retrieval, *K*_m_ value prediction [32] and capturing protein changes [33, 34]. Although these methods may not be highly explanatory, they have significantly enhanced accuracy. However, a notable gap remains: machine learning has not yet been deployed for enzyme-sequence screening of given substrate-product pairs.

**Figure 1:**
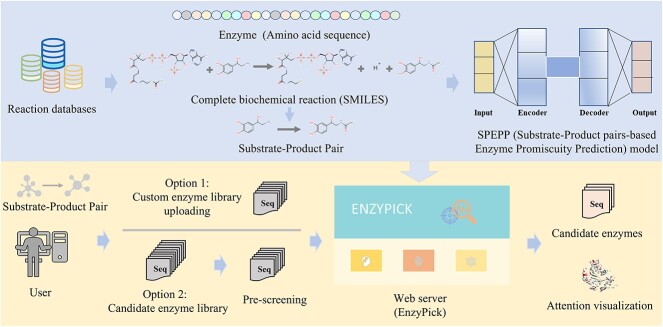
Overview of the Substrate–product Pair-based Enzyme Promiscuity Prediction (SPEPP) model and EnzyPick. EnzyPick, our web-based platform built on the SPEPP model, streamlines enzyme screening and analysis without requiring deep-learning expertise. Users can upload their enzyme libraries and obtain reaction likelihood scores. For those without custom libraries, we provide pre-screening options. EnzyPick provides enzyme sequences and scores, as well as atom-to-atom mapping results and enzyme structure visualizations. SMILES: Simplified Molecular Input Line Entry System.

To address the gap, we introduce Substrate–product Pair-based Enzyme Promiscuity Prediction (SPEPP) model, a deep-learning model for enzyme screening that outputs a score indicating the possibility that an enzyme catalyzes a substrate–product reaction. Our methodology involves the collection of substrate–product–enzyme triads from existing reaction databases, generating negative data based on EC numbers, and applying transformers and transfer learning techniques to construct the model. The SPEPP model stands apart from existing methods in its independence from the EC number system. This independence facilitates a more expansive and accurate appraisal of candidate enzymes, encompassing a myriad of reactions and substrates hitherto uncharted by conventional EC-centric methodologies. Furthermore, it can provide a reference for enzyme modifications based on attention weights. Crucially, SPEPP allows users to incorporate candidate-enzyme libraries from any source; this flexibility considerably broadens the scope of enzyme screening. The SPEPP model is versatile, lending itself to many biological scenarios involving substrate–enzyme-product relationships, ranging from enzyme screening for biosynthesis pathway design and degradation of hazardous materials to product or substrate screening. To further extend the reach and usability of our model, we developed EnzyPick, a web server platform for enzyme screening based on the SPEPP model (Figure 1). EnzyPick is available at http://www.biosynther.com/enzypick/.

## MATERIALS AND METHODS

### Dataset construction

**Figure 2:**
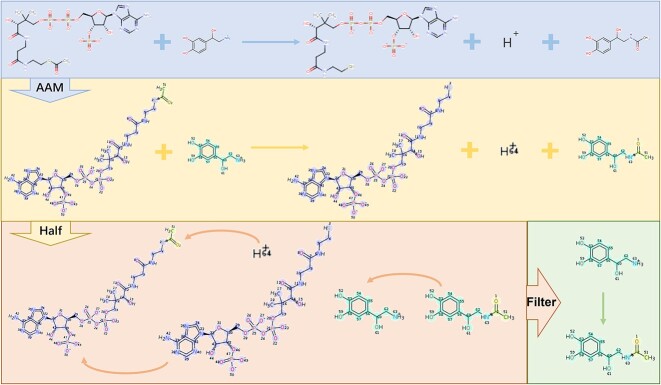
Atom-to-atom mapping-based extraction of substrate–product pairs from metabolic pathway-related complete reaction data in existing databases. AAM: atom-to-atom mapping. For the remaining details, see the Methods section, Dataset construction.

This study required substrate–product–enzyme triads derived from reaction data. Comprehensive reaction data for metabolic pathways were sourced from various databases, including KEGG [35], Rhea [36], BRENDA [37], MetaCyc [38] and RxnFinder [39]. Construction involved an atom-to-atom mapping technique using the Reaction Decoder Tool (RDT) [40]. By inputting the complete reaction into the RDT, we determined the atom-to-atom correlation between the substrate and product, thereby identifying viable substrate–product pairs. These pairs were selected based on the criterion that >50% of the atoms constituting product C were derived from substrate A (Figure 2; Half). In biochemical reactions, cofactors such as ATP and ADP are ubiquitously present. Given the focus of our study on predicting enzyme specificity in catalyzing substrate-to-product conversions, molecules like ADP, ATP and H+ are not primary reactants but rather commonly act as cofactors. Failing to eliminate these compounds from our analysis could potentially introduce biases into the model’s predictions. Therefore, we enacted a data-cleaning procedure, removing substrate–product pairs composed of single atoms (e.g. H^+^) and excluding cofactors such as ATP and ADP (Figure 2, Filter). The resulting substrate–product pairs were then paired with the corresponding enzymes from the original reactions to obtain the necessary substrate–product–enzyme triads. Furthermore, enzymes with sequences >1000 amino acids in length were excluded from the dataset to circumvent GPU memory limitations and enhance training efficiency.

To mimic the ratio of positive-to-negative examples found in real-world scenarios while ensuring a balanced dataset for each model-training iteration, we generated unlabeled samples at 20 times the volume of positive samples. The method for generating these negative examples was as follows: for a reaction *R* with corresponding enzymes *E1*, *E2*, …, *En* with a four-digit EC number, any enzyme with a different EC number was labeled as ‘unlabeled.’ These unlabeled enzymes are unlikely to catalyze reaction *R*. To balance the number of negatives against memory constraints, 20 unlabeled enzymes were randomly selected for each reaction.

### Undersampling learning

Most of the unlabeled enzymes, which were treated as negatives during training, were indeed true negatives. However, to address the potential inclusion of positives within the unlabeled data, we incorporated a regularization technique, label smoothing [41], to temper the model’s predictions and reduce overfitting. Label smoothing is central to calculating the loss function. Our chosen loss function was a binary cross-entropy loss and label smoothing hybrid. The mathematical representation of the fusion function is as follows: 


(1) \begin{equation*} L=-\frac{1}{N}\sum_{i=1}^N\left[\left(\epsilon \bullet \mathrm{y}\right)\log (p)+\left(1-\left(1-\epsilon \right)\bullet \mathrm{y}\right)\log \left(1-p\right)\right] \end{equation*}

where $N$ is the batch size, $\mathrm{y}$ is the ground truth, $p$ is the predicted possibility, $\epsilon$ is the label smoothing rate, and $\epsilon$ was set to 0.1.

To ensure a balanced dataset during training, we randomly selected the same number of negatives from the unlabeled data as there were positives. This approach deviates from traditional training methods by employing a random selection strategy for balanced training, matching the number of negative examples in the unlabeled pool with the number of positive examples. This dynamic changed when the model exhibited a receiver operating characteristic area under the curve (ROC–AUC) > 0.9, a threshold indicative of high performance. Subsequently, unlabeled instances with a predicted value >0.8 were purged from the training data, ensuring they did not contribute to further training iterations.

### Data feature extraction

Word2Vec [42] was used to process enzyme-sequence features. Every three amino acids were encoded, and the feature vector length was set to 100 [43], resulting in *N* × 100 enzyme-sequence features [44]. The hidden layer output vector, the output layer vector, and the conditional probability of the output were calculated as follows: 


(2) \begin{equation*} {\displaystyle \begin{array}{rr}& h=\frac{1}{C}{W}^Tx\\[3pt] {}& y= Uh\\[3pt] {}& p\left({w}_O|{w}_I\right)=\frac{\exp \left({y}_{w_O}\right)}{\sum_{w=1}^V\exp \left({y}_w\right)}\end{array}} \end{equation*}

where $C$ is the number of context amino acids (AAs) (set to 2); $x$ is the input vector of an AA; $V$ is the vocabulary size (set to 21); $W$ is the first weight matrix, of size $V\times N$; $N$ is the number of word-embedding dimensions (set to 100); $h$ is the hidden layer output vector (size $N\times 1$), which can be used as the feature vector for the context AAs; $U$ is the second weight matrix, of size $N\times V$; $y$ is the output layer vector, of size $V\times 1$; $p\left({w}_O|{w}_I\right)$ is the conditional probability of the output AA ${w}_O$ given the input context AAs ${w}_I$; and ${y}_{w_O}$ and ${y}_w$ are the elements of $y$ corresponding to ${w}_O$ and $w$, respectively.

To identify substrate–product pair features, we first used RXNFP [45], a BERT-based [46] model for extracting features from complete chemical reactions, but it can handle imbalanced ones to obtain features for substrate–product pairs. In this model, ${\mathcal{D}}_c$ and ${\mathcal{T}}_c$ are the source domain and task for chemical reaction prediction from RXNFP, respectively, and ${\mathcal{D}}_b$ and ${\mathcal{T}}_b$ are the target domain and task for biochemical substrate–product–enzyme triad prediction, respectively. Transfer learning was then used to improve the learning of $P\left({Y}_b|{X}_b\right)$ in ${\mathcal{D}}_b$ with the information gained from ${\mathcal{D}}_c$ and ${\mathcal{T}}_c$, where ${\mathcal{D}}_b\ne{\mathcal{D}}_c$ or ${\mathcal{T}}_b\ne{\mathcal{T}}_c$. To adapt RXNFP for this task, we froze the parameters for all network layers and appended two convolutional layers and a linear layer to further extract the features.

### Model construction

Transformer architecture [47], selected because of its efficacy across a range of applications, formed the foundation of our model. To enhance feature extraction, we modified the standard Transformer by eliminating the positional encoding information from the encoder and decoder and substituting the fully connected layers with convolutional layers. In the encoder, a multi-head self-attention mechanism was adopted to assign individual weights to each element of the input protein sequence.

The decoder comprises two multi-head attention layers: the first processes substrate–product–enzyme triads, and the second identifies the interaction between the pair and the protein sequence. After processing via a feedforward network, the encoder output is introduced into the attention mechanism. Within the decoder, a self-attention mechanism is then applied to the substrate–product pair features. Subsequently, cross-attention is applied between the resulting data and the encoder output. Ultimately, the output is produced via a fully connected Softmax layer.

To initialize the weights, we used fixup initialization [48], a method developed to ensure that the output from each residual block maintains unit variance. This technique helps to prevent gradient explosions and supports the training of deeper models. For each layer $l$ in a residual block, the weight matrix ${W}_l$ is initialized as follows: 


(3) \begin{equation*} {\displaystyle \begin{array}{rr}& {W}_l\sim \mathcal{N}\left(0,\frac{\sigma_l}{\sqrt{n_l}}\right)\\{}& {\sigma}_l=\left\{\begin{array}{ll}\sqrt{2}& \mathrm{if}\ {l}_r=1\\{}\sqrt{0.5}& \mathrm{if}\ {l}_r={n}_r\\{}1& \mathrm{if}\ 1<{l}_r<{n}_r\end{array}\kern0.5em \right.\end{array}} \end{equation*}

where ${\sigma}_l$ is a scaling factor that depends on the layer type and position; ${n}_l$ is the number of input units to the layer; and ${l}_n$ is the ${l}_n^{th}$ layer in each residual block.

The attention mechanism in SPEPP can be described as follows: 


(4) \begin{equation*} {\displaystyle \begin{array}{rr}& Attention\left(Q,K,V\right)=\frac{\mathit{\exp}\left(\frac{Q{K}^T}{\sqrt{d}}\right)}{\sum_i\mathit{\exp}\left(\frac{Q{K}^T}{\sqrt{d}}\right)}V\\{}& \alpha \left(q,k\right)=\mathit{\exp}\left({q}^Tk/\sqrt{d}\right)\\{}& \alpha \left(q,{k}_i\right)=\frac{\alpha \left(q,{k}_i\right)}{\sum_{j=1}^n\alpha \left(q,{k}_j\right)}\\{}& f\left(q,\left({k}_1,{v}_1\right),\left({k}_2,{v}_2\right),\dots, \left({k}_n,{v}_n\right)\right)=\alpha \left(q,K\right)V\end{array}} \end{equation*}

where $q\in{R}^{d_q}$ is the query; $k\in{R}^{d_k}$ is the key; $v\in{R}^{d_v}$ is the value; and *Q*, *K*, and *V* are the mini-batch representations of *q*, *k*, and *v*. Multi-head attention was defined as 


(5) \begin{equation*} \kern0.5em {h}_{\iota }= Attention\left({W}_{\iota}^qq,{W}_{\iota}^kk,{W}_{\iota}^vv\right) \end{equation*}

where ${h}_{\iota }$ is the $\iota$^th^ head in multi-head attention, and ${W}_{\iota}^q\in{R}^{p_q\times{d}_q}$, ${W}_{\iota}^k\in{R}^{p_k\times{d}_k}$ and ${W}_{\iota}^v\in{R}^{p_v\times{d}_v}$ are learnable parameters.

The model was constructed utilizing PyTorch 1.12 (https://pytorch.org/). We set the number of epochs to 64, set the batch size to 64, the learning rate to 5E-6, the weight decay to 1E-5 and the drop rate to 0.1. We configured the number of layers to 12, the number of attention heads to 8, the hidden dimensions, and the norm shape of the model to 64. We used an improved Adam [49] named Radam [50] as the optimizer. For the training, four NVIDIA Tesla V100 32GB GPUs were utilized. The training process lasted approximately 14 days.

### Statistical tests

Given the extensive size of the dataset (1 030 882 positives and 19 234 075 negatives), the conventional data splitting method (training data: test data, 8:2) was not appropriate. After removing duplicate data, we randomly selected 5000 positives and 5000 negatives for analysis.

To evaluate model performance, ROC-AUC and precision–recall curve (PRC)-AUC were used as evaluation metrics. In computational biology, ROC-AUC and PRC-AUC are commonly used metrics for evaluating the performance of classification models, especially in scenarios where classification tasks are predominant [51]. These metrics are computed using the following equations:


(6) \begin{equation*} {\displaystyle \begin{array}{rr}& TPR=\frac{TP}{TP+ FN}\\[3pt] {}& FPR=\frac{FP}{FP+ TN}\\[3pt] {}& Precision=\frac{TP}{TP+ FP}\\[3pt] {}& Recall=\frac{TP}{TP+ FN}\\[3pt] {}& ROC- AUC={\int}_0^1 TPR(FPR)\ dFPR\\[3pt] {}& PRC- AUC={\int}_0^1 Precision(Recall)\ dRecall\end{array}} \end{equation*}

where *TP * refers to true positives, *FP* to false positives, *TN* to true negatives, and *FN* to false negatives. The ROC-AUC curve represents the true positive rate (*TPR*) versus the false positive rate (*FPR*) at various threshold settings. PRC-AUC quantifies the overall ability of the classification model to discriminate between positive and negative classes. The precision–recall curve depicts the trade-off between precision and recall for various threshold settings. PRC-AUC, the total area under this curve, is robust against class imbalance.

### Web server

The EnzyPick web server was created using Python on Ubuntu 18.04.2. The implementation adheres to the latest web standards, including HTML 5 and CSS 3. The Bootstrap Web (https://getbootstrap.com/) frameworks was used to create the front-end interface. Backend development was conducted in Python and Django application frameworks. To visualize a protein in Protein Data Bank format on a Django web server, we used a JavaScript library named NGL.js [52]. Chemical molecules and reactions are depicted using SmilesDrawer.js [53].

## RESULTS

### Data acquisition and negative data generation

SPEPP is a deep-learning model based on transfer learning. The data in this work comprised substrate–product–enzyme triads gleaned from comprehensive reaction datasets in extant databases (Methods, Dataset construction) via atom-to-atom mapping (Figure 2). Due to the constraints imposed by GPU memory limitations, enzymes exceeding 1000 AAs in length (7538 of 204 942) were deleted from the dataset; the final dataset had 197 404 enzymes.

Because negatives are seldom cataloged in databases, their acquisition poses a significant challenge. To mirror real-world proportions of positives to negatives while maintaining the dataset balance required for each model-training epoch, we adopted a novel approach based on the premise that an enzyme arbitrarily selected from a non-target EC number is unlikely to catalyze a reaction in the same manner as the target EC number. We deviated from the conventional 1:1 positive-to-negative sample ratio by generating unlabeled samples at a scale 20 times larger than that of positive samples. This approach resulted in 1 030 882 positive and 19 234 075 negative substrate–product pairs. After eliminating duplicates, we randomly segregated 5000 positives and 5000 unlabeled data points to constitute the test set, with the remaining data forming the training set. Notably, 89.3% of the sequences in the test set exhibited average sequence identity (SI) [54] below 30% when compared with the sequences in the training set. The average SI for the test dataset was 24.56%. For each sequence in the test set, on average, 99.16% of the other sequences along with the sequence itself had SI lower than 30%. The training set had an average SI of 24.34%. For each sequence, on average, 99.24% of the other sequences along with the sequence itself had an SI below 30%.

### Deep-learning model: Construction and performance

**Figure 3:**
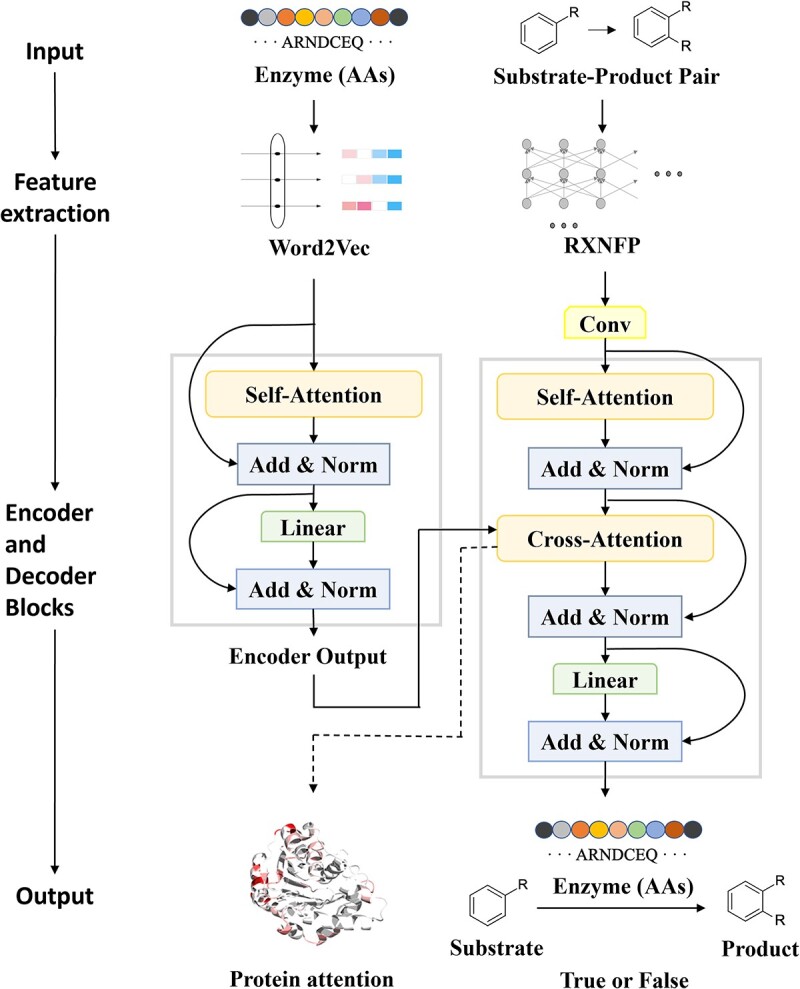
The architecture of the SPEPP model, with Word2Vec, RXNFP-based transfer learning, and multi-head attention mechanisms. For a more streamlined mapping of attention weights directly to enzymes, we used the classic Word2Vec instead of large pre-trained models such as ESM-2. A multi-headed self-attention mechanism is employed in the encoder to assign distinct weights to each element in the input protein sequence, enabling nuanced extraction of enzyme features. RXNFP was used to encode substrate–product pairs. The decoder has two multi-headed attention layers: the first layer processes information from the substrate–product pair, while the subsequent layer processes the interaction between the pair and the enzyme. The final output is derived through a fully connected Softmax layer.

We established an effective model-training workflow that optimizes the undersampling-learning and data-balancing strategies (Figure 3). SPEPP employs RXNFP [45]-based transfer learning to process substrate–product pairs.

The model then uses the multi-head attention mechanism, which is grounded in the transformer architecture, to process substrate–product pairs and enzymes. The undersampling-learning strategy of our model was meticulously refined to enhance the training process. SPEPP demonstrated remarkably good performance, with a high ROC–AUC of 0.993 and a PRC–AUC of 0.994 for the test data. We performed additional model validation using a cleaned dataset from reference [55], ensuring it lacked any training set data. Using the methods described earlier, we converted the reaction–enzyme pairs in this dataset into substrate–product–enzyme triads and constructed a negative dataset. We randomly selected an equivalent number of negative samples to maintain a balanced test dataset. After purging the data that overlapped with the model-training dataset, we obtained a final dataset of 963 positives and 963 negatives. SPEPP maintained its high performance in zero-shot [56], accurately identifying 83.021% (734 of positives, 865 of negatives) of the 1926 instances.

To benchmark our approach against existing models, we subjected the dataset to ESP [57], the SOTA model at the moment. We split the 1926 substrate–product–enzyme triads into 1926 enzyme-substrate pairs and 1926 enzyme-product pairs and then uploaded them to the website of ESP (https://esp.cs.hhu.de/ES_pred_multiple). Our evaluation strategy was heavily biased in favor of ESP. Specifically, as long as one of the two predictions (the enzyme–substrate pair and the enzyme-product pair) is correct, the result is deemed correct. Under this criterion, the accuracy of ESP model was 66.20% (correctly identifying 319 out of 963 positives and 956 out of 963 negatives). However, if both predictions were required to be correct, the accuracy of ESP remarkably decreased to 49.94% (with correct identification of 67 out of 963 positives and 895 out of 963 negatives).

### Using SPEPP for enzyme screening in large-scale datasets

In enzyme screening, the conventional approach hinges on leveraging prior knowledge to deduce the reaction type, followed by the use of an EC number query to compile a list of potential enzymes. However, this strategy can fail when faced with an expansive list of thousands of enzymes with the same EC number and without clear criteria for their prioritization. Our method is a screening tool that assigns each candidate enzyme a likelihood score for its catalytic potential, thus facilitating its ranking. Notably, this model can screen enzymes from user-defined custom libraries.

**Figure 4:**
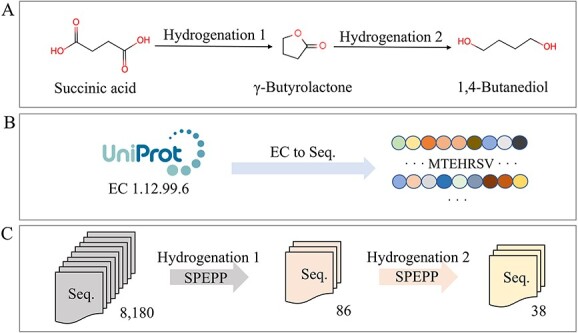
Demonstration of screening for enzymes involved in the conversion of succinic acid to 1,4-butanediol. (**A**) Hydrogenation reactions with succinic acid and 1,4-butanediol as the substrate and product, respectively. (**B**) Enzyme-sequence screening of all 8180 EC 1.12.99.6 sequences from UniProt as of December 2023. (**C**) Two different substrate–product pairs are involved in the conversion of succinic acid to 1,4-butanediol. SPEPP screened all 8, 180 sequences pair by pair. Finally, SPEPP screening identified 38 shortlisted sequences.

To demonstrate the potential of our model, we applied SPEPP to a substrate–product pair, succinic acid and 1,4-butanediol (Figure 4A). The current reduction reaction from succinic acid to 1,4-butanediol primarily employs chemical methods or multiple-step biochemical reactions [58, 59]. Succinic acid undergoes two hydrogenation steps to produce 1,4-butanediol [58]. We explored the possibility of a single enzyme catalyzing both hydrogenation steps and conducted the following experiments: Given the hydrogenative nature of this reaction, we selected all hydrogenases listed under EC 1.12.99.6 in the UniProt database as of December 2023, resulting in a pool of 8180 enzymes (Figure 4B). After two rounds of hydrogenation enzyme screening, only 38 enzymes remained (Figure 4C). Importantly, each remaining enzyme had a reference value that aided in ranking.

**Figure 5:**
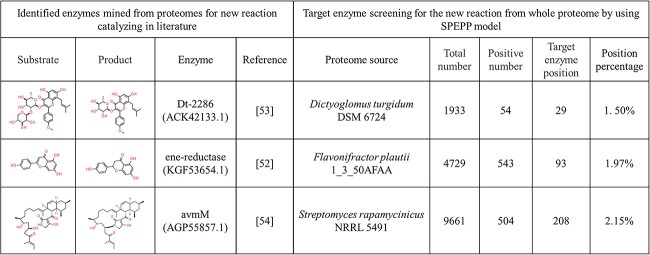
Examples of enzyme screening for novel reactions from whole proteomes using the SPEPP model. The information in the first four columns is from the sources cited. ‘Total number’: count of proteins in the proteome. ‘Positive number’: count of proteins predicted as positive by the SPEPP model. ‘Target enzyme rank’: SPEPP-derived ranking of the named enzyme. ‘Relative rank’: the enzyme’s rank relative to the total number of enzymes in the proteome.

We further validated the screening process by applying it to the complete proteomes of species containing target enzymes to identify enzymes capable of catalyzing recently discovered substrate–product pairs [60–62]. After sourcing the complete proteomes, we fed the substrate–product pair and protein data from references (Figure 5, Reference) into our model. The target enzymes consistently ranked within the top 3% of the proteins in the proteome (Figure 5). This highlights the potential of the SPEPP model for large-scale dataset-based screening of enzymes. Yang *et al*.[60] discovered an ene-reductase that initiates flavone and flavonol catabolism in gut bacteria by employing a blend of bioinformatics, biochemicals, and genetic analyses. Without any specific analysis, the SPEPP model ranked this target protein 93^rd^ among the 4774 proteins just by using the proteins of the gut microbe proteome as the input. Similarly, from a pool of 1993 proteins from *Dictyoglomus turgidum* (DSM 6724) proteome, our model ranked the newly identified β-xylosidase/α-arabinosidase/β-glucosidase Dt-2286, which catalyzes the conversion of Sagittatoside B into Baohuoside I [61], in the 29^th^ place. The model also successfully identified AvmM, which mediates macrocyclization via dehydration/Michael-type addition in Alchivemycin A biosynthesis, among the top 2% of the 9661 proteins in the *Streptomyces* sp. TP-A0867 proteome [62]. These cases exemplify how SPEPP can swiftly screen candidate enzymes from large datasets for user-defined custom libraries, bypassing the need for extensive and intricate bioinformatic analyses. Beyond its applications in enzyme selection for pathway design and mining, SPEPP also offers flexibility in various substrate–product–enzyme -related scenarios, including substrate or product screening with a given enzyme.

### Development of EnzyPick to facilitate SPEPP utilization

**Figure 6:**
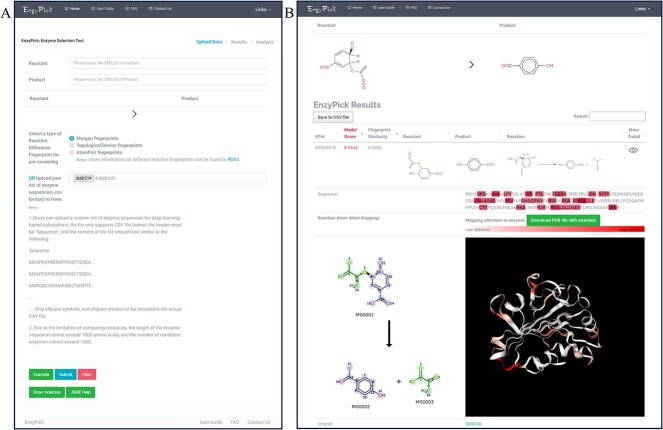
EnzyPick web interface screenshots. (**A**) EnzyPick homepage, including inputs for the reactant and product and the option to upload a custom enzyme library or select a pre-screening method for the candidate-enzyme library. (**B**) EnzyPick results page, displaying the enzyme sequences and enzyme-catalyzed scores, as well as comprehensive visualization tools, including atom-to-atom mapping and attention-score mapping.

To extend the utility of our model, we developed EnzyPick (http://www.biosynther.com/enzypick/), an online web server based on the SPEPP model (Figure 6A). EnzyPick integrates data, model functionality, and visualization tools, facilitating comprehensive screening and analysis.

EnzyPick allows users to upload custom enzyme libraries, enabling the model to predict enzyme-catalyzed reactions directly. Based on the user-provided enzyme library, the server produces possibility scores for potential downstream tasks (Figure 6B). The server provides a library as a viable option for users without pre-prepared candidate-enzyme libraries. We implemented a pre-screening step that condenses the selection scope to expedite screening. Further, the server provides a candidate-enzyme pre-screening method based on similarity metrics, namely Morgan fingerprints [63], Topological Torsion fingerprints [64] and Atom-Pair fingerprints [65]. Among them, Morgan fingerprints depict molecular structure through the connectivity of atoms in local environments [66]. Topological Torsion fingerprints encode the 3D arrangements using atom quadruples, and Atom-Pair fingerprints represent molecules based on the types and distances between atom pairs. In terms of results analysis, EnzyPick goes beyond merely presenting the enzyme sequence and possibility scores for enzyme-catalyzed reactions; the platform also delivers atom-to-atom mapping results for reference reactions linked to specific enzyme sequences [67], as well as enzyme structure visualizations equipped with attention-weight mapping (Methods, Deep-Learning Model Architecture). This additional information is invaluable for subsequent post-selection enzyme modification (Figure 6B).

## CONCLUSIONS AND DISCUSSION

Identifying functional enzymes for catalyzing specific reactions has a pivotal role in enzyme engineering and application. Traditionally, enzyme screening has been based on substrate–product screening, which involves testing the ability of different enzymes to convert a given substrate into a desired product. Despite its ubiquity, this approach is labor-intensive and time-consuming and may overlook potential enzymes with specificity for the target reaction. Hence, a more efficient and precise enzyme-screening method is needed to harness the wealth of enzyme data available across databases and the literature.

To address this, we developed a deep-learning model to predict whether an enzyme is capable of catalyzing a specific substrate–product pair. Unlike traditional methods that rely on known reactions as reference points and compare candidate enzymes’ structural or functional similarities with reference enzymes, our method does not require prior knowledge of reactions, enabling users to curate candidate-enzyme libraries from any source. This method uses a deep neural network to elucidate the complex relationships between enzyme features and substrate–product pairs and returns a score representing the possibility of an enzyme catalyzing the substrate–product pair.

The SPEPP model has several advantages over existing methods. Based on the speed and scalability arising from its lack of computationally expensive similarity comparison steps, it can screen thousands of enzymes in minutes, using only CPU resources, making it ideal for high-throughput screening. Additionally, the versatility of our model extends beyond the tasks described in this article, and it can address any substrate–product–enzyme task. EnzyPick thus has potential applications in designing and optimizing biocatalytic systems, finding alternative substrates or products for a given enzyme, and discovering novel reactions for a given substrate or product. Furthermore, it can recognize novel enzymes with high potential to catalyze target reactions based on their features.

The current embedding model utilized by SPEPP for enzyme sequence processing is Word2Vec, selected for its ability to map the resultant vectors onto the enzyme sequences. This mapping facilitates correlating SPEPP’s attention scores with specific enzyme sites, underscoring the importance of particular regions within the sequence. The correlation is unachievable with some methods, such as PSSM [68], and while one-hot encoding could theoretically serve this purpose, its high sparsity and limited capacity to capture the relational nuances of AAs renders it unsuitable.

Regarding large-scale pre-trained models such as ESM-2 [69], these models can associate input protein sequences with their own attention scores. However, using these models as embedding models results in fixed-dimensional vectors, for instance, ESM-2’s 1280-dimensional vector and 640-dimensional vector. Such vectors cannot be mapped to individual AAs in the enzyme sequence, which in turn disrupts the correspondence between the model’s attention scores and specific enzyme sites.

Notably, the training set size for large-scale pre-trained models exceeds that of the enzymes involved in this project significantly. Hence, employing such models for embedding—without concentrating on attention mapping—might yield superior results. We plan to explore this potential, particularly focusing on how to map attention scores with enzyme sequences when using pre-trained models.

In the future, we intend to enhance the diversity and coverage of our dataset by incorporating more enzyme data from diverse sources (databases, literature and new experiments), potentially augmenting the generalizability of the SPEPP model. We aim to enhance model performance by optimizing the model parameters and experimenting with cutting-edge models, potentially better capturing the nuances of enzyme and substrate–product pairs and improving model accuracy and robustness. Moreover, we aim to extend its functionality to other tasks related to enzyme engineering, including predicting reaction mechanisms or kinetics for enzyme-substrate–product triads or designing novel enzymes for a given substrate–product pair, potentially contributing to advances in enzyme engineering.

In summary, we propose a deep-learning enzyme-screening method based on substrate–product pairs, addressing critical issues in enzyme engineering. The model’s effectiveness was demonstrated using several benchmark datasets, and it outperformed existing methods in terms of screening range, accuracy, and speed. We also provide examples of the application of our method to various substrate–product enzyme tasks. We anticipate that our method will ease the process of enzyme screening for catalysis and synthesis and lead to advances in enzyme engineering.

**Key Points**

- The SPEPP model is designed for enzyme promiscuity prediction.
- It effectively highlights the interactions between enzymes and substrate–product pairs.
- Uniquely, it can predict functions beyond the scope of the Enzyme Commission system.
- EnzyPick, built on the SPEPP framework, is tailored to assist researchers lacking programming skills.
- It boasts high-throughput capabilities and empowers users to define candidate-enzyme libraries.

## Data Availability

EnzyPick is freely available at http://www.biosynther.com/enzypick/. The data used in this study were sourced from various databases. All data are publicly available. However, in some cases, user licenses are required to access the underlying data. The dataset of substrate-product pairs for model training was collected from Rhea (https://www.rhea-db.org/), KEGG (https://www.genome.jp/kegg/), MetaCyc (https://metacyc.org/), RxnFinder (http://www.rxnfinder.org/rxnfinder/), and Brenda (https://www.brenda-enzymes.org/). To facilitate further usage, the detailed instructions and all codes for model training and testing are provided in a Zenodo repository: https://doi.org/10.5281/zenodo.8210150. Any additional information required to reanalyze the data reported in this paper is available from the corresponding author upon reasonable request.

## Author Biographies

**Huadong Xing** is a Ph.D. candidate at the Shanghai Institute of Nutrition and Health, Chinese Academy of Sciences (CAS). His research interests are in deep learning and computational biology.

**Pengli Cai** is a research associate at the Shanghai Institute of Nutrition and Health, CAS. Her research interests include microbial cell factory design and biosynthesis.

**Dongliang Liu** is a Ph.D. candidate from the Shanghai Institute of Nutrition and Health, CAS. His research interests include deep learning and computational biology.

**Mengying Han** is a Ph.D. candidate from the Shanghai Institute of Nutrition and Health, CAS. Her research interests include pathway design and deep learning.

**Juan Liu** is a professor at the Institute of Artificial Intelligence, School of Computer Science, Wuhan University. Her group is focusing on machine learning, data mining, intelligent computing and bioinformatics.

**Yingying Le** is a professor at the Shanghai Institute of Nutrition and Health, CAS. Her group is focusing on metabolic homeostasis regulation and disorders-related diseases.

**Dachuan Zhang** is a researcher at ETH Zurich. His research interests include bioinformatics, machine learning, and life cycle assessment.

**Qian-Nan Hu** is a professor at the Shanghai Institute of Nutrition and Health, CAS. His group focuses on deep learning, synthetic biology, computational chemistry, and reaction pathway design.
